# Cognitive Profile in Schizophrenia With and Without Alcohol Use Disorder: Domain-Specific and Global Effects (BACS/WCST)

**DOI:** 10.1192/j.eurpsy.2026.10452

**Published:** 2026-07-31

**Authors:** M. S. Artemieva, E. Koshkin, I. Danilin, T. Mkrtchyan

**Affiliations:** Peoples’ Friendship University of Russia named after Patrice Lumumba, Moscow, Russian Federation

## Abstract

**Introduction:**

Alcohol use disorder (AUD) is common in schizophrenia and may aggravate neurocognitive dysfunction; however, domain-level effects remain inconsistent. Clarifying domain patterns and composite behaviour may improve clinical profiling and rehabilitation in dual diagnosis.

**Objectives:**

To compare cognition in schizophrenia with comorbid AUD (SchAl) versus schizophrenia without AUD (Sch) across BACS subtests, the BACS composite, and WCST, and to provide clinically oriented interpretation.

**Methods:**

Fifty-four adults with schizophrenia in stable remission were assessed: Sch (n=28) and SchAl (n=26). Cognition was measured with the Brief Assessment of Cognition in Schizophrenia (BACS) and the Wisconsin Card Sorting Test (WCST). BACS scores were standardised to local norms (z). Group comparisons used the Mann–Whitney U-test (two-tailed, p<0.05). All were assessed during abstinence/remission; intoxication or withdrawal were exclusionary. Qualitative notes captured fatigability, rule-clarification requests, and early termination.

**Results:**

Sch outperformed SchAl in verbal learning (38.6±7.61 vs 33.7±5.49; p=0.024) and digit sequencing (17.6±2.80 vs 15.7±1.53; p=0.006). Executive functions were poorer in SchAl: Tower of London (15.1±2.78 vs 13.3±2.10; p=0.018), and WCST perseverative errors were higher (39.1±7.61 vs 43.9±6.75; p=0.043). Processing-speed subtests were largely comparable: Coding and Verbal Fluency were non-significant, whereas Sch showed an advantage on Token Motor (61.6±17.12 vs 55.9±17.19; p=0.045). Importantly, the BACS composite favoured Sch (221.9±33.0) over SchAl (201.2±28.9; p=0.006), indicating a broad cognitive burden associated with AUD comorbidity beyond isolated subtests. Observationally, SchAl more often exhibited fatigability, slower initiation, rule-clarification requests, and premature task cessation; Sch occasionally produced overly high move counts on Tower of London but corrected after feedback.

**Conclusions:**

In schizophrenia, comorbid AUD is associated with clinically meaningful deficits in verbal and working memory and executive control, higher perseveration on WCST, and a lower BACS composite, while processing speed is mostly preserved apart from motor speed. The pattern suggests selective vulnerability of maintenance/manipulation and planning under dual diagnosis, with relative sparing of basic perceptual–motor speed. Clinically, these findings support integrated care and domain-targeted cognitive remediation (working memory, planning, set-shifting), alongside monitoring of treatment adherence and fatigue management. Larger longitudinal cohorts with adjustment for antipsychotic exposure, smoking, and mood symptoms are warranted to refine mechanisms and inform personalised rehabilitation.

**Disclosure of Interest:**

None Declared

